# Pontellid copepods, *Labidocera* spp., affected by ocean acidification: A field study at natural CO_2_ seeps

**DOI:** 10.1371/journal.pone.0175663

**Published:** 2017-05-03

**Authors:** Joy N. Smith, Claudio Richter, Katharina E. Fabricius, Astrid Cornils

**Affiliations:** 1 Australian Institute of Marine Science, Townsville, Queensland, Australia; 2 Alfred Wegener Institute Helmholtz Centre for Polar and Marine Research, Bremerhaven, Germany; 3 University of Bremen, Bremen, Germany; Helmholtz-Zentrum fur Ozeanforschung Kiel, GERMANY

## Abstract

CO_2_ seeps in coral reefs were used as natural laboratories to study the impacts of ocean acidification on the pontellid copepod, *Labidocera* spp. Pontellid abundances were reduced by ∼70% under high-CO_2_ conditions. Biological parameters and substratum preferences of the copepods were explored to determine the underlying causes of such reduced abundances. Stage- and sex-specific copepod lengths, feeding ability, and egg development were unaffected by ocean acidification, thus changes in these physiological parameters were not the driving factor for reduced abundances under high-CO_2_ exposure. *Labidocera* spp. are demersal copepods, hence they live amongst reef substrata during the day and emerge into the water column at night. Deployments of emergence traps showed that their preferred reef substrata at control sites were coral rubble, macro algae, and turf algae. However, under high-CO_2_ conditions they no longer had an association with any specific substrata. Results from this study indicate that even though the biology of a copepod might be unaffected by high-CO_2_, *Labidocera* spp. are highly vulnerable to ocean acidification.

## Introduction

Copepods are microscopic crustaceans that dominate most seawater and freshwater zooplankton communities [1,2], from the tropics to the poles [3]. They have a wide range of morphologies and behaviors [4], and play an important ecological role in aquatic food chains. Within the marine realm, copepods are also vital to the microbial loop, remineralization of nutrients, and the biological pump [5,6]. Because copepods are a crucial link between phytoplankton primary producers and higher trophic levels, any changes in copepod populations may disseminate throughout entire marine ecosystems.

Anthropogenic carbon dioxide emitted into the atmosphere gets absorbed by surface waters in the ocean and changes its chemistry [7,8]. The addition of carbon dioxide limits the amount of available carbonate ions in the water column and reduces seawater pH, in a process called ocean acidification (OA) [9–11]. Lowered aragonite and calcite saturation states under OA reduce calcification [8,12,13], thus initial OA research on plankton primarily focused on calcifying taxa like coccolithophores and pteropods [14–17]. In recent years, effort has been extended to also understanding OA impacts on copepods [18–22]. The exoskeletons of copepods are composed of chitin [23], a modified polysaccharide containing nitrogen. Chitin contains no calcium carbonate and is therefore considered unresponsive to OA. Nonetheless, the sheer abundance and importance of copepods to global ocean ecosystems makes understanding their reaction to changes in seawater chemistry indispensable.

To date, the effect of OA on planktonic copepod species worldwide is poorly understood. In part this is due to the high diversity of marine copepods (>2,000 species described to date [24]), with various species likely responding differently to the same stress. The initial consensus was that copepods are mostly tolerant to OA [25–27], although recent evidence has begun to challenge this viewpoint [28].

Multigenerational studies on copepods under OA conditions suggest that naupliar production declines [21], juveniles are often more sensitive than the adults [29], metabolic costs increase [30], and reproductive success becomes limited [31]. Copepods exposed for short experimental periods to OA conditions are often more negatively impacted than copepods that have been exposed to OA for a second generation [32]. The ability of copepods to tolerate changes in seawater pH is also highly associated with the natural range of environmental conditions they live in [33,34]. Additional research indicates that OA may alter the nutritional quality of copepod prey, which has negative consequences for copepod somatic growth and egg production [35]. Furthermore, changes in nutritional quality can reduce the trophic transfer efficiency of carbon from phytoplankton to copepods [36], although changes in the phytoplankton caused by OA do not always have a negative impact on copepods [37]. Combining all the research on how copepods may cope with OA shows that the answer is quite complex. Responses are likely species-specific, with several species expected to fare well under OA, and both direct and indirect impacts affecting copepods simultaneously [38].

Most studies thus far on copepods have been conducted in the laboratory and on generalist species that are naturally tolerant to a wide range in environmental parameters and laboratory conditions. Laboratory experiments provide valuable information on understanding the underlying mechanisms of how OA affects the copepods, however few copepod species have been studied to date, and no single species has been studied for its response to OA in its natural environment. The study presented here examines OA effects on a copepod species in the field in its natural environment. Furthermore, it focuses on non-generalist copepods adapted to a narrow range of environmental conditions under the assumption that it may be less tolerant to change, including OA, than generalist species that live in a wide range of conditions. We conducted this field study at natural CO_2_ seep sites in coral reefs where copepods live residential within their natural habitat. Reef-associated zooplankton are able to maintain their position within reefs [39], by living amongst the seafloor substrata [40], swimming against currents [41], and swarming behind corals to avoid being swept away by currents [42]. Residential zooplankton live locally within the reef and, therefore, those copepods residing at the high-CO_2_ reefs have presumably been exposed to OA their entire lifetime, and likely for multiple generations.

Although *Labidocera* copepods are traditionally considered as neustonic, some species live residentially within coral reefs [43]. Residential pontellids were reduced in abundance at coral reefs exposed to high-CO_2_ conditions compared to ambient conditions, when examined at the family level [28]. Furthermore, Pontellidae were more sensitive to OA compared to other zooplankton [28]. Due to their apparent sensitivity to OA, we chose to study the *Labidocera* pavo species group (consisting of one dominant and two very similar infrequent species with almost identical morphology and biology) and at different life stages, to understand the effects of OA on their biology. This study had the following objectives: 1) Determine the effects of OA on total abundances as well as for each life stage for copepodites C2-C5 and adults in *Labidocera* spp., 2) Determine if aspects of their biology, specifically stage-specific copepod length, gut content, and egg development, were affected by OA, and 3) Determine if their associations with day-time reef substrata were affected by OA.

## Methods

### Study site

**Fig 1:**
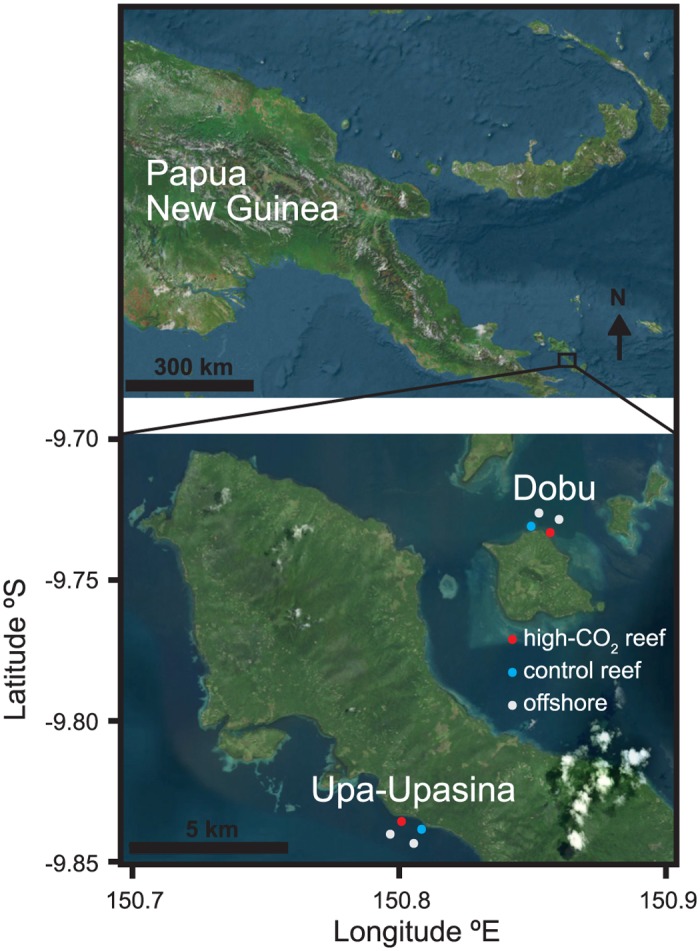
Map of study sites at Dobu and Upa-Upasina in Papua New Guinea. Blue circles indicate control sites over the reef, red circles indicate high-CO_2_ sites over the reef, and white circles indicate offshore sites.

The effects of ocean acidification on *Labidocera* spp. were examined at two separate CO_2_ seeps and adjacent control sites (Dobu and Upa-Upasina) in Milne Bay Province, Papua New Guinea (Fig 1). The distance between high-CO_2_ and control sites for both Dobu and Upa-Upasina is approximately 500 m, with control sites along the same fringing reef as the high-CO_2_ reefs but outside the influence of the CO_2_ seeps. By geodesic distance, Dobu and Upa-Upasina are ∼10 km apart and separated by Dobu and Normanby Islands, and are completely separate volcanic seeps. The seeps release ∼99% CO_2_ gas into fringing coral reefs, locally reducing seawater pH. The higher *p*CO_2_ and associated changes in the carbonate chemistry parameters are the only differences in seawater chemistry between the seeps and the adjacent control sites [44]. Water temperature (27–29°C) and salinity (∼34.5 psu) are similar along the CO_2_ gradients, and so are geomorphology and oceanographic parameters of the study sites. Two Nortek 1 MHz AWACs (Acoustic Wave and Current meters) and two Kongsberg ADCPs (Acoustic Doppler Current Profilers) were deployed continuously, one of each instrument at both the control and high-CO_2_ reef sites. Depending on the tide, water depths were between 2–3 m at both the control and high-CO_2_ sites. Furthermore, at both Dobu and Upa-Upasina, water flowed along the shore with current speeds < 5 cm s^-1^, switching directions with diurnal tides. Thus, the oceanographic conditions were similar between the control and high-CO_2_ sites at both Dobu and Upa-Upasina.

Copepods were collected at control (averaged pH_T_ = 8.0) and high-CO_2_ sites (averaged pH_T_ = 7.8) at the Dobu and Upa-Upasina seeps and their associated control reefs, and for two expeditions (24 May–9 June 2013, and 22 March–17 April 2014) while onboard the M/V Chertan. For each night, three replicate horizontal tows were collected at both the control and high-CO_2_ sites. During the first expedition, Dobu was sampled for 2 consecutive days (i.e. sample number = 6 per CO_2_ site), Upa-Upasina for 8 days (n = 24), and during the second expedition Dobu was sampled for 3 days (n = 9) and Upa-Upasina for 6 days (n = 18). Additionally during both expeditions, horizontal net tows were conducted offshore from the high-CO_2_ and control sites at water depths of 50–70 m in order to compare abundances between high-CO_2_ reefs and control reefs to offshore waters.

The carbonate chemistry at the study sites has been documented previously and exhibits diurnal and ephemeral fluctuations [28,44–46]. Long-term seawater pH averages were calculated from continuous measurements by deployed SeaFET ocean pH sensors and discrete water samples measurements that were collected along spatial and temporal gradients, fixed with mercuric chloride solution, and later analyzed for the dissolved inorganic carbon and total alkalinity using a Versatile Instrument for the Determination of Total Inorganic Carbon and Titration Alkalinity. Dissolved inorganic carbon and total alkalinity were used to calculate other seawater carbonate chemistry parameters, including pH at total scale using the Excel macro CO2SYS [47]. Seawater at the high-CO_2_ seep sites has an average pH of 7.8, the pH level expected for the end of the century if carbon dioxide emissions continue unabated [48]. Thus, the reef-associated copepods living residential to the high-CO_2_ reefs in this study, including *Labidocera* spp., are living in ocean acidification conditions, and the insight into their biology from these CO_2_ seep sites may help predict their outcome in future oceans.

### Sample collection

Papua New Guinea’s Department of Environment and Conservation Marine Scientific Research Committee granted permission to conduct research in D’Entrecasteaux Islands, Milne Bay Province. No copepods collected in this study are listed as endangered or protected species. Copepods were collected at night using horizontal net tows and emergence traps. Three replicate horizontal net tows were collected per night at both the control and high-CO_2_ sites between 2100–0200 hours over several consecutive nights at both seeps and during both expeditions. Each tow was along a 30 m transect parallel to the shoreline using a Nansen net (70 cm aperture diameter, 100 μm mesh size) at a speed of approximately 1 knot. The tows were conducted in shallow water (2–3 m depth) with the plankton net approximately 1 m above the reef. A Hydro-Bios digital flowmeter was attached to the center of the net aperture to record the exact volume of the water sampled.

Under ocean acidification conditions at these seep sites in Papua New Guinea, the dominant substrate shifts from complex branching corals to bouldering corals [44]. To investigate if this shift in dominant substrata had an impact on *Labidocera* spp. abundance, we first assessed what the preferred substrata were of these particular copepods. A substrata preference experiment was conducted over 10 days in total during the second expedition at the Upa-Upasina reef: 5 days at the control site to determine their substrate associations under normal CO_2_ condition, and 5 days at the high-CO_2_ site to determine if these substrate associations changed under ocean acidification conditions, with the control and high-CO_2_ sites being sampled on alternating nights within the 10 day period. Nine emergence traps were deployed each night with three replicates over the three dominant substrate types (coral rubble, branching coral, and bouldering coral). 'Dominant' was defined as >50% cover by the given type of substratum. In total, 45 traps were sampled per CO_2_ treatment.

The emergence traps were pyramid-shaped 1 m tall ‘tents’ made of 100 μm plankton mesh attached to a 1x1 m^2^ quadrat, following the design of Porter and Porter (1977) [49]. Detachable cod-ends that contained a weak light (3 lumens) were attached to the top of the pyramid. The traps were deployed during the day between 1500–1700 hours when few zooplankton were present in the water column. Cod-ends were collected at night between 2000–2100 hours, after the demersal copepods emerged into the water column after dusk (~18:30). Emergence traps were placed over three dominant substrata types (coral rubble, branching coral, and massive bouldering coral) in random different locations around the reef each day. Since no quadrat was covered 100% by any one substratum type, photos were taken of each quadrat and the percent coverage of the three dominant and non-dominate substrata (sand, fleshy macro algae, and turf algae) were estimated.

All samples were preserved in 4% formalin buffered with sodium borate and stored for further analysis.

### Laboratory analysis

Samples from both the horizontal tows and emergence traps were divided in half using a Folsom splitter, and *Labidocera* spp. abundances were counted in half of the original sample using microscopy. Additionally, *Labidocera* spp. collected during the second expedition were enumerated by life stage (copepodite stages 2–5 [C2-C5] and adults). Males and females were identified separately for copepodite C5 and adults. The youngest life stages were not counted since they were too small to be caught with the 100 μm mesh of the plankton net. The same copepods enumerated by life stage were also measured for their total length to determine if size differences may occur under OA.

Individual females were randomly selected from each sample (5–15 individuals per sample) across all days sampled at the high-CO_2_ and control sites and from both Dobu and Upa-Upasina sites. In total, 248 females from the horizontal tows were examined for their gut fullness and the maturity of their oocytes. Each individual adult female copepod was dissected under the microscope to determine the oocyte developmental stages according to the classification of Niehoff (2003) [50]. The gonad morphology of *Labidocera* spp. matched the description of the *Acartia*-type gonad [51], where all oocyte developmental stages are present. In our case, all females carried many small immature oocytes in their ovaries and diverticula, and thus, we only marked the females carrying also mature oocytes, i.e. large oocytes with visible nuclei or of irregular shape, that occur prior to spawning and indicate that final oocyte developmental processes take place [50].

To compare feeding ability, the guts of the 248 female specimens were dissected. It was noted whether the guts of the female copepods were empty, 1/3 full, 2/3 full, or completely full. Compact fecal pellets were only rarely observed.

### Statistical analysis

All statistical analyses were computed in R version 3.2.2 (R Development Core Team, 2016). Generalized linear models (GLMs) with a quasipoisson distribution and log link function were used to determine the effects of CO_2_, reef, and expedition on *Labidocera* spp. abundance on total abundances, abundances of each life stage, and the number of mature oocytes inside the adult females. GLMs with a gaussian distribution were used to determine effects of CO_2_ and reef on total length for each life stage. GLMs with a quasibinomial distribution were used to determine the effects of CO_2_ and reef on gut fullness. GLMs with a poisson distribution and log link function were used to determine the effects of date and the percent coverage of each substratum type (coral rubble, branching coral, bouldering coral, turf algae, macro algae, sand) on *Labidocera* spp. abundance in the emergence traps at the control and high-CO_2_ sites. Model assumptions of independence, homogeneity of variance, and normality of error were evaluated through diagnostic tests of leverage, Cook’s distance, and dfbetas [52]. Checks for all GLMs indicated that no influential data points or outliers existed in the data and model assumptions were met.

## Results

Four species of *Labidocera* were present in the samples, with a strong dominance by *L*. *bataviae* (~70% of *Labidocera* specimens). *L*. *pavo*, *Labidocera* sp. (a yet un-described new species), and *L*. *laevidentata* were the other species identified. The latter was morphologically different from the other three species [53], rare (<1%) and was therefore excluded from further analysis. However, *L*. *bataviae*, *L*. *pavo*, and *Labidocera* sp. are closely related and belong to the *pavo* species group within the *L*. *detruncata* species complex [54]. These three species are considered to have similar behaviors and have the same size ranges; their copepodites are morphologically identical, and in the adults only the shape of the 5^th^ swimming leg and the urosome is different [54]. Thus, for this study *Labidocera* spp. represents the three species *L*. *bataviae*, *L*. *pavo*, and the un-described species *Labidocera* sp., corresponding to 99% of the total *Labidocera* specimens collected in the samples. Furthermore, Labidocera comprised between 43–100% of the total pontellids present, which included the genera *Calanopia*, *Pontella*, and ‘other’.

### Reduced abundances for later life stages under high-CO_2_ conditions

**Fig 2:**
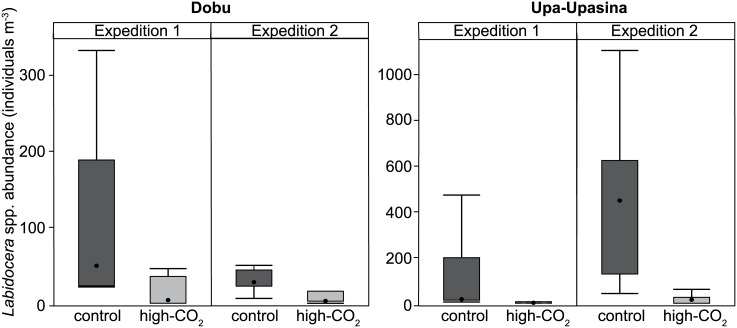
Differences in *Labidocera* spp. abundance between CO_2_ sites and reefs. Values for two expeditions, at two reefs (Dobu and Upa-Upasina) under control and high-CO_2_ conditions. The box represents the median, first and third quartiles and the whiskers represent the minimum and maximum values.

Total abundances of *Labidocera* spp. were highly reduced at the high-CO_2_ sites (F_(1,112)_ = 76.8, p < 0.001; Fig 2), in spite of the also significant differences in abundance between reefs (F_(1,111)_ = 15.4, p < 0.001), expeditions (F_(1,110)_ = 10.2, p = 0.002), and the interaction between reef and expedition (F_(1,107)_ = 5.1, p = 0.027). Two-way interaction terms (CO_2_:reef and CO_2_:expedition) had no significant influence on total *Labidocera* spp. abundance (F_(1,109)_ = 0.69, p = 0.410 and F_(1,108)_ = 0.13, p = 0.714), and the three-way interaction term (CO_2_:reef:expedition) was also non-significant (F_(1,106)_ = 1.2, p = 0.274).

*Labidocera* spp. abundances were higher at the high-CO_2_ reefs compared to offshore waters where water depth was 50–70 m and reefs were absent (Table 1). This difference in abundance confirmed a large proportion of the *Labidocera* spp. were resident to the seeps, supporting the observation that they live residential to coral reefs.

**Table 1:** Abundances and standard deviations of *Labidocera* spp. at each study site.

Reef	Expedition	Site	N	Mean abundance (ind. m^-3^)	STD
Dobu	1	reef at high-CO_2_	6	14.86	20.33
		reef at control	6	110.70	125.75
		offshore from high-CO_2_	3	0	n/a
		offshore from control	3	0	n/a
	2	reef at high-CO_2_	9	22.52	36.10
		reef at control	9	31.27	14.32
		offshore from high-CO_2_	3	2.76	2.40
		offshore from control	3	2.86	0.20
Upa-Upasina	1	reef at high-CO_2_	24	55.75	140.91
		reef at control	24	163.83	284.16
		offshore from high-CO_2_	8	0.56	0.60
		offshore from control	8	0.71	0.79
	2	reef at high-CO_2_	18	16.80	18.99
		reef at control	18	371.20	344.94
		offshore from high-CO_2_	5	7.79	5.94
		offshore from control	5	5.75	6.74

**Fig 3:**
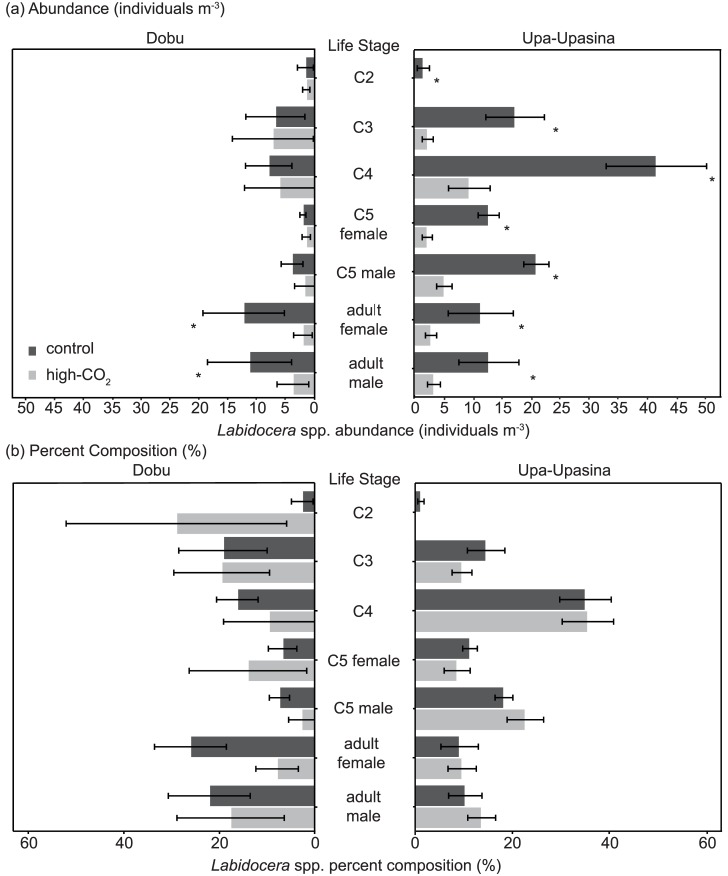
Abundance and percent composition of juvenile and adult life stages of *Labidocera* spp. under ocean acidification. (A) Abundance (number m^-3^) of copepodites (C2-C5) and adult *Labidocera* spp. at control and high-CO_2_ sites for Dobu and Upa-Upasina reefs. (B) Percent composition (%) of copepodites (C2-C5) and adult *Labidocera* spp. at control and high-CO_2_ sites for Dobu and Upa-Upasina reefs. (*) indicates a significant difference between the control and high-CO_2_ conditions for each life stage.

The abundance of each life stage was examined in the samples from the second expedition. At Upa-Upasina, abundances of all life stages were significantly reduced under ocean acidification. At Dobu only adult abundances (males and females) were reduced while abundances of the younger stages were unaffected by ocean acidification conditions (Fig 3A). There was no difference in abundance between control and high-CO_2_ sites for copepodite C2 (F_(1,16)_ = 2.8, p = 0.119), which were quite rare in the samples (2% of individuals). Furthermore, there were no differences in the percent composition of each life stage within the total *Labidocera* spp. community between CO_2_ levels or reefs (Fig 2B). The ratio between copepodites to adults was also not different between CO_2_ levels (F_(1,16)_ = 0.9, p = 0.368), but it was different between reefs (F_(1,15)_ = 7.0, p = 0.019), while the interaction between CO_2_ and reefs was non-significant (F_(1,14)_ = 0.1, p = 0.819). Also, the ratio of males to females was unaffected by all parameters, CO_2_ (F_(1,16)_ = 0.01, p = 0.937), reef (F_(1,15)_ = 0.4, p = 0.531), and there was no interaction between the two factors (F_(1,14)_ = 0.6, p = 0.443).

### Copepod biology unaffected by ocean acidification

#### Copepod lengths unaffected by OA

**Fig 4:**
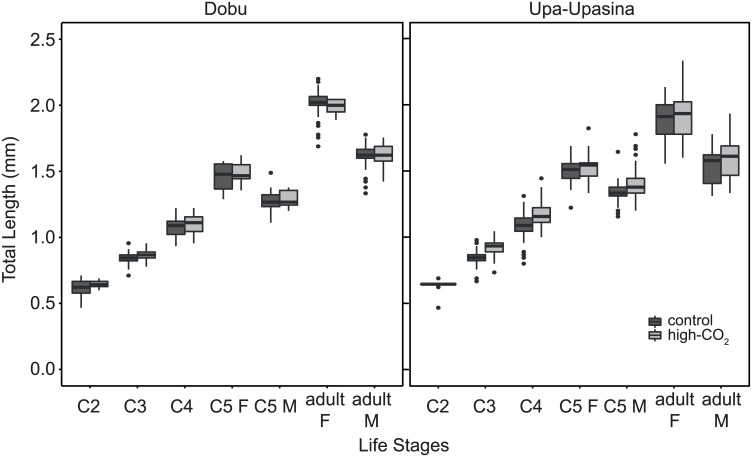
Difference in length of life stages of *Labidocera* spp. between CO_2_ levels for Dobu and Upa-Upasina reefs. Total length for copepodites (C2-C5) and adult copepods. The lengths are compared between control and high-CO_2_ conditions for each life stage, and results are presented for Dobu and Upa-Upasina reefs. The box represents the median, first and third quartiles and the whiskers represent the minimum and maximum values.

Each of the life stages have the same size ranges across the three *Labidocera* species [55], justifying a pooling of the data of all three species for the length analyses. There was no difference in copepod lengths between high-CO_2_ and control sites for the adult males, the adult females, or the copepodite life stages, or between CO_2_ levels within a life stage (Fig 4).

#### No difference in gut fullness under OA

**Fig 5:**
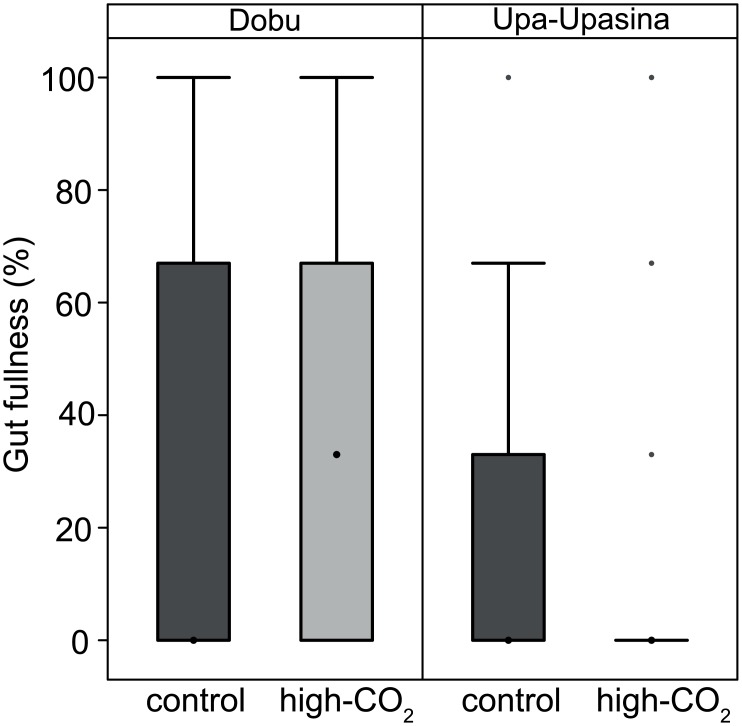
Gut fullness of *Labidocera* spp. at Dobu and Upa-Upasina reefs at control and high-CO_2_ sites. The box represents the median, first and third quartiles and the whiskers represent the minimum and maximum values.

There was no difference in the gut fullness of adult female copepods between CO_2_ sites (χ^2^ = 114, df = 152, p = 0.20), but gut fullness differed between reefs (greater gut fullness at Upa-Upasina then at Dobu reef (χ^2^ = 356, df = 151, p = 0.02). CO_2_ differences were not present even when Upa-Upasina reef was evaluated separately (χ^2^ = 48, df = 71, p = 0.40, Fig 5).

#### No difference in the number of oocytes under OA

**Fig 6:**
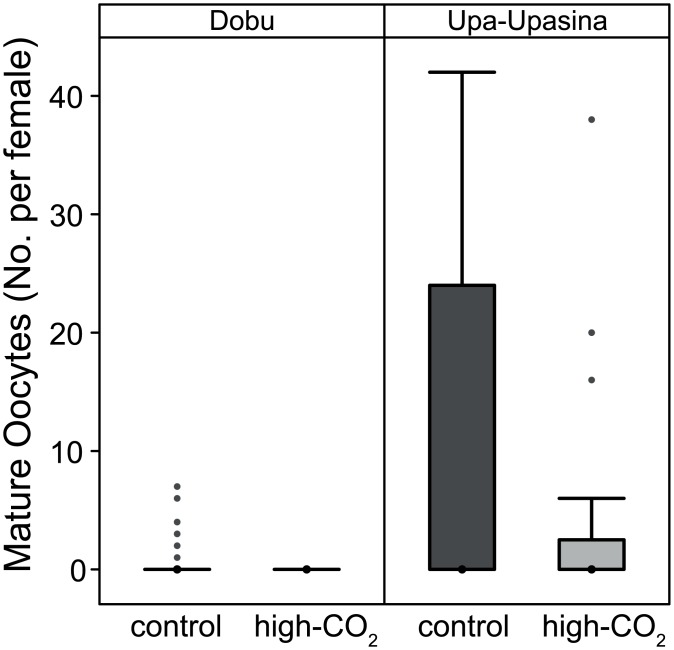
Number of mature oocytes in adult female *Labidocera* spp. at Dobu and Upa-Upasina reefs at control and high-CO_2_ sites. Number of mature oocytes inside the diverticula of adult female copepods. The box represents the median, first and third quartiles and the whiskers represent the minimum and maximum values.

Immature oocytes in the ovaries and the diverticula were present in all females, but not all females had mature gonads. Thus, only the occurrence and number of mature oocytes were noted in adult females. The number of mature oocytes in the adult females copepods was not different between CO_2_ levels (χ^2^ = 20, df = 152, p = 0.18), but copepods at Upa-Upasina had more mature oocytes than those at Dobu (χ^2^ = 614, df = 151, p < 0.01; Fig 6).

### No substrate association under ocean acidification

**Fig 7:**
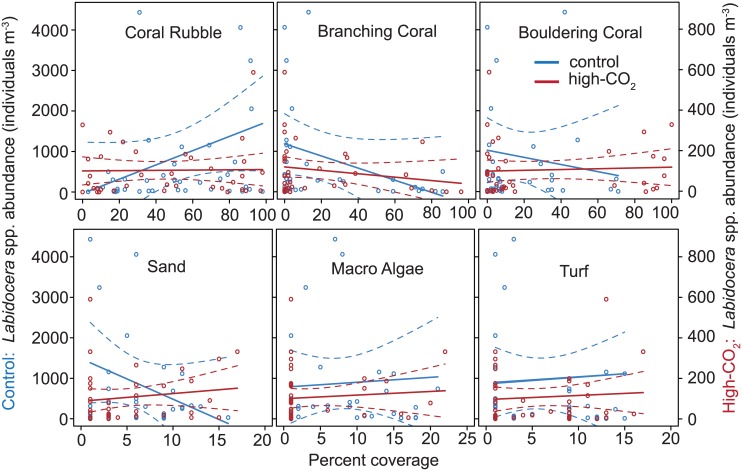
*Labidocera* spp. abundances and substrata cover at high-CO_2_ and control sites of Upa-Upasina. Copepod abundance (number m^-3^) as a function of percent cover of coral rubble, branching coral, bouldering coral, sand, macro algae and turf for control and high-CO_2_ sites at Upa-Upasina reef.

Emergence traps data showed a reduction in *Labidocera* spp. abundance at the high-CO_2_ site over all types of substrata. At the control reef, their abundances were significantly associated with the cover of coral rubble, macro algae, and turf algae (Fig 7). In contrast, at the high-CO_2_ site, their abundances were not correlated with any specific substratum (Fig 7; Table 2). Instead, their numbers were consistently low for all substrata. Furthermore, F values declined for all substrates under high-CO_2_ conditions compared to the controls. In particular, the F value for turf algae at the control was 87.8 times the F value at the high-CO_2_ sites (turf algae: F-value_control_ = 7.9 vs F-value_high-CO2_ = 0.09). Similarly, for macro algae and coral rubble F values at the control were 3.6 and 146.7 greater than at the high-CO_2_ sites, respectively (macro algae: F-value_control_ = 4.7 vs F-value_high-CO2_ = 1.3; coral rubble: F-value_control_ = 4.4 vs F-value_high-CO2_ = 0.03). Overall, associations with substrata at the control sites weaken and nearly disappear under high-CO_2_ conditions. Aside from substrata, the abundance of *Labidocera* spp. differed according to the date sampled (date: F-value_control_ = 22.6 vs F-value_high-CO2_ = 2.7). Despite natural fluctuations from day to day sampling, *Labidocera* spp. was consistently reduced at the high-CO_2_ sites for all substrata.

**Table 2:** Effect of date and percent cover of reef substrata on *Labidocera* spp. abundance at control and high-CO_2_ sites.

		Control		High-CO_2_	
Parameter	(df,df)	F	p	F	p
Date	4,26	22.6	<0.001	2.7	0.053
% Turf Algae	1,30	7.9	0.009	0.09	0.755
% Macro Algae	1,31	4.7	0.040	1.3	0.258
% Coral Rubble	1,35	4.4	0.044	0.03	0.872
% Branching Coral	1,34	3.3	0.082	2.2	0.147
% Massive Bouldering Coral	1,33	3.7	0.064	0.15	0.699
% Sand	1,32	3.6	0.067	0.13	0.719

## Discussion

Our field study examining the effects of ocean acidification on the pontellid copepod *Labidocera* spp. showed reductions in total abundances and in the abundances of in most life stages and in both sexes (copepodite C3-C5 and adult life stages). Volcanic CO_2_ seeps create conditions to study *in situ* changes to OA for fully acclimatized groups of organisms in their natural habitat, i.e., under natural levels of food and substratum availability, predation, currents, temperature and light, and unaltered capacity for nocturnal migration. Our results were consistent across two separate seep sites, and over two expeditions. We have shown before that total abundances of zooplankton residing in coral reefs may be reduced in response to OA, with some species-specific differences in the severity of responses between taxa [28]. Here we show that for the pontellid copepods, abundances of all life stages were reduced under high-CO_2_ at Upa-Upasina, while only adults but not younger life stages were reduced at Dobu. We also show that reductions in *Labidocera* spp. abundances were not due to changes in stage-specific sizes, feeding (gut fullness), or reproduction (oocyte numbers). In contrast, our data suggest that under future OA conditions, these copepods no longer associate with a specific substratum type.

None of the parameters measured, including copepod length, gut fullness, and the number of mature oocytes, were affected by ocean acidification. Growth is often measured on individual copepods from start to end of an experiment [56], or from length-weight ratios [56]; both methods were not suitable for this field study as it is unknown how copepod weights compare between CO_2_ levels and we did not measure feeding within a specific time frame to calculate feeding rates. Instead, we measured copepod lengths for hundreds of individuals at all life stages from both the control and high-CO_2_ sites, and found that their lengths remained unaffected by CO_2_ (Fig 4).

Gut fullness is an indicator of rates of feeding, food assimilation and egestion [57,58]. The similar levels of gut fullness between the control and high-CO_2_ sites suggested that these three factors were unaffected by OA. Most laboratory experiments examine feeding rates between a start and end time point under exposure to different CO_2_ levels. Laboratory experiments on copepod feeding rates under ocean acidification have shown mixed results [27,30,59], with grazing of some species unaffected by high-CO_2_ and other species increasing their feeding rates [60]. Logistic constraints precluded the execution of incubation experiments to measure feeding rates. However, we measured gut fullness, which is an estimate of their ability to feed, which was unimpaired for *Labidocera* spp. Thus, bottom-up constraints from consuming different quantities of food are unlikely to explain the reduced abundances found at the high-CO_2_ reefs.

The quantity of food found in the guts of *Labidocera* spp. remained unaltered under OA, but perhaps changes in their diet may have contributed to their reduced abundances. *Labidocera* spp. are omnivorous, consuming phytoplankton and small zooplankton [61,62]. Phytoplankton biomass did not differ between the control and high-CO_2_ sites, and the quality of phytoplankton is also assumed to be similar between CO_2_ levels [28]. However, the abundance of other zooplankton taxa, including smaller copepods like Paracalanidae that *Labidocera* spp. may feed on [63], is reduced at the high-CO_2_ seeps sites [28]. Thus, *Labidocera* spp. may rely more on phytoplankton for food if copepod prey density is reduced. Changes in diet would likely (but not necessarily) result in changes in growth or reproduction [64,65], however neither were affected by high-CO_2_ conditions. Therefore, any repercussions of potential changes in their diet should be explored to further understand possible bottom-up influences on *Labidocera* spp. abundances.

When comparing the control reefs to the high-CO_2_ reefs, top-down influences from increased predation on *Labidocera* spp. are an unlikely explanation for reduced abundances at the high-CO_2_ sites. In fact, predation of these pontellid copepods by other zooplankton, planktivorous fish, and corals at the seeps is likely to be less at the high-CO_2_ reefs compared to the control sites. Within the zooplankton community, the larger zooplankton taxa that would prey on these copepods were also highly reduced at the seep sites [28]. Furthermore, nocturnal planktivorous fish that would primarily feed on the nocturnally migrating *Labidocera* spp. were also highly reduced in abundance at these seep sites, although the diurnal planktivous fish abundance remained the same under OA [66]. Corals also capture and consume zooplankton, and *Galaxea fascicularis* showed preferential consumption of pontellid copepods. Yet, their feeding rates declined in high-CO_2_ conditions; thus, predation at least from some coral species will be reduced. Much of the pontellid predators in the reef are also reduced because they, too, are also affected by the shift in habitat complexity from branching corals to bouldering corals, leading to an overall decrease in top-down pressure on copepod abundance.

Differences in predation are also an unlikely explanation for the differences in abundance between offshore and over the reef. Planktivorous fish form ‘walls of mouths’ and deplete zooplankton near coral reefs [67]. In comparison to other zooplankton taxa, *Labidocera* spp. are large in size and therefore have few plankton predators, although it cannot be ruled out that some meroplankton (e.g. crab larvae) would prey on them. In offshore waters, *Labidocera* spp. predators (meroplankton, planktivorous fish, and corals) are highly reduced or absent. Despite a greater presence of predators on the reef, *Labidocera* spp. abundances are greater over the reef compared to offshore waters, which is further supporting evidence for them living residential to the reef.

Multigenerational studies suggest that egg production can be either suppressed [29] or unaffected by elevated CO_2_ [30], depending on the copepod species. As *Labidocera* spp. are residential to the reef [43], they are assumed to be exposed to ocean OA conditions for the majority of their lifetime, and likely for multiple generations. Isolated islands often have endemic species of coastal zooplankton suggesting they have successful retention mechanisms [68]; nonetheless, nothing is known about the connectivity of these copepods between reefs, or whether these copepods self-recruit as do some demersal marine organisms [69], or if they disperse as nauplii. Thus, total exposure time to high-CO_2_ conditions is unknown, but all life stages starting from copepodite C2 through to adults were consistently found more abundantly over the reef and not offshore, suggesting that most of their lives are spent residential to the reef and exposed to ocean acidification conditions near the seeps. Despite the long-term exposure to high-CO_2_, we observed that OA did not have an apparent effect on the number of oocytes produced within the oviducts of the adult females. However, nothing is known about hatching success rates or the quality of the oocytes (i.e. yolk formation, of which *Labidocera* copepods have three distinct forms of endogenous yolk [70]).

Results are mixed as to whether juvenile copepods are more affected by ocean acidification compared to the adults. Some studies show no effect on juvenile copepods [26,71], while others reveal naupliar production is reduced and that juveniles are less likely to survive than the adults [19,21]. Although we did not collect nauplii nor copepodite stage I (C1), we could compare the ratios between copepodites (stages C2-C5) to adult abundances since copepodites may still be vulnerable to OA [30], and the ratio was unaffected by high-CO_2_ conditions. However, since naupliar stages are often the most vulnerable, we cannot rule out the possibility that a full analysis including the ratio of nauplii to adults may have shown a different result. Thus, an investigation in hatching success and nauplii survival under high-CO_2_ conditions at the seep sites is an important next step to further understand the underlying causes for reduced abundances at the high-CO_2_ reefs.

Nearly all ocean acidification experiments conducted in the laboratory use females, thus very little is known of how males react to CO_2_ stress compared to the females [20,26,71]. Our study showed that the ratio between males and females remained unaffected by CO_2_, therefore both sexes appeared equivocally impacted by ocean acidification.

Although there were no differences in copepod length, gut fullness, and oocyte production between high-CO_2_ and control sites, these measures all differed between reefs. Lengths of each stage were slightly larger at Upa-Upasina than at Dobu, and female adults had more food in their stomachs and a larger number of mature oocytes at Upa-Upasina compared to Dobu. Increased feeding at Upa-Upasina likely explains why the copepods at Upa-Upasina reef were slightly larger and had more energy available to generate oocytes than at Dobu.

Changes in habitat from branching coral to more massive bouldering coral explains why some zooplankton taxa are reduced at these seep sites [28]. That does not seem to be the case for *Labidocera* spp. whose preferred day-time habitat is coral rubble, macro algae, and turf algae. Importantly, these three substrata types have similar percent cover across the high-CO_2_ and control sites, with coral rubble and macro algae covering ~3% and ~5%, respectively. Turf algae had an almost equal percent cover at the high-CO_2_ and controls sites (~35% vs 38%). This suggests that this genus of copepods loses their association with specific reef substrata. At the high-CO_2_ sites, *Labidocera spp*. abundances were low at all types of substrata, and unrelated to the percent coverage of each substratum.

How or why *Labidocera* spp. lose their ability to associate with a substratum type is unknown, but perhaps OA affects the chemical sensory ability of copepods to detect where to live in the reef. Copepods have light receptors, mechanosensory setae, chemosensory sensilla, and bimodal sensilla that are all used to detect physical and chemical cues within their environment [72]. Ocean acidification disrupts the ability of some tropical coral reef fish species to recognizing reef substrata as home [73,74]. Nothing is known about copepods’ ability to smell coral reefs, but considering the vital role of olfaction in copepods to detect mates, food, and predators [72], it is likely that it may also play an important role to help *Labidocera* spp. smell a suitable substratum. Similarly, some meroplankton species use smell in addition to other cues (e.g. sound [75], vision [76]) to detect and settle on their preferred substrata in coral reefs [77,78]. It therefore remains to be explored whether there is a disruption in the sensory capabilities of *Labidocera* spp. in high-CO_2_ conditions to smell their preferred substrate within the reef, or a change in the smell released by the substrata.

There are other potential explanations for the observed reduction in abundances, including a potential avoidance of high-CO_2_ areas. In a flume laboratory choice experiment, the copepod *Centropages tenuiremis* preferred to stay in seawater of ambient pH 8.15 or slightly reduced pH (7.8), and avoided seawater with low pH levels of 7.6 and 7.0 [79]. Note that at a pH of 7.8, which was the condition at our high-CO_2_ sites, *C*. *tenuiremis* did not avoid the CO_2_-enriched seawater. Complete avoidance is unlikely since otherwise there would be no *Labidocera* spp. present at the high-CO_2_ sites; however, further investigations should determine if *Labidocera* spp. may avoid high-CO_2_ seawater. The ability of *Labidocera* spp. to smell or taste their preferred substrate, as well as high-CO_2_ seawater, should be studied in order to understand the underlying mechanisms behind *Labidocera* spp. abundance loss at the reefs under ocean acidification conditions.

The results of this study highlight a few important points relevant to OA research on copepods. First, a dramatic reduction within the community of certain sensitive species, like *Labidocera* spp., suggests that such species may be indicator species for habitats impacted by ocean acidification. For example, if the pontellid abundances begin to decline, it may be a signal that the zooplankton community is being affected by environmental changes. Second, the field results suggest conclusions about OA tolerances derived from laboratory studies may be unsubstantiated. For *Labidocera* spp., the field results indicate that although many aspects of their biology may be unaffected by ocean acidification conditions, their populations may still be vulnerable to OA, as their abundances were reduced under high-CO_2_. Third, this is the first study to suggest that the ability of these copepods to detect their preferred habitat may be compromised. If these copepods can no longer detect their home, or simply avoid it, their abundances may be reduced via reduced survival. This study indicates the importance of combining field observations with field and laboratory experiments to understand how OA may impact copepods and other marine organisms in a future high CO_2_ world.

Copepods living in the open ocean where substrata preference is not relevant will not face the same problems, but understanding the mechanisms why *Labidocera spp*. no longer express an association with specific substrata may be relevant for other copepods and should be further investigated. If the chemoreception of copepods was compromised under OA, this could also impact oceanic copepods, which too use smell for a number of important biological purposes. Laboratory experiments should therefore be conducted on *Labidocera* spp. to determine why they are not found associated with their preferred reef substrata at near-future levels of elevated CO_2_.
